# A mixed methods multiple case study of implementation as usual in children’s social service organizations: study protocol

**DOI:** 10.1186/1748-5908-8-92

**Published:** 2013-08-20

**Authors:** Byron J Powell, Enola K Proctor, Charles A Glisson, Patricia L Kohl, Ramesh Raghavan, Ross C Brownson, Bradley P Stoner, Christopher R Carpenter, Lawrence A Palinkas

**Affiliations:** 1 Brown School, Washington University in St. Louis, Campus Box 1196, One Brookings Drive, St. Louis, MO 63130, USA; 2 College of Social Work, University of Tennessee, Knoxville, TN 37996, USA; 3 Department of Psychiatry, Washington University School of Medicine, St. Louis, MO 63130, USA; 4 Department of Surgery and Siteman Cancer Center, Washington University School of Medicine, St. Louis, MO 63130, USA; 5 Department of Anthropology, Washington University in St. Louis, St. Louis, MO 63130, USA; 6 Department of Internal Medicine, Division of Infectious Diseases, Washington University School of Medicine, St. Louis, MO 63130, USA; 7 Department of Emergency Medicine, Washington University School of Medicine, St. Louis, MO 63130, USA; 8 School of Social Work, University of Southern California, Los Angeles, California 90089, USA

**Keywords:** Implementation strategies, Mental health, Children and adolescents, Mixed methods, Multiple case study

## Abstract

**Background:**

Improving quality in children’s mental health and social service settings will require implementation strategies capable of moving effective treatments and other innovations (*e.g*., assessment tools) into routine care. It is likely that efforts to identify, develop, and refine implementation strategies will be more successful if they are informed by relevant stakeholders and are responsive to the strengths and limitations of the contexts and implementation processes identified in usual care settings. This study will describe: the types of implementation strategies used; how organizational leaders make decisions about what to implement and how to approach the implementation process; organizational stakeholders’ perceptions of different implementation strategies; and the potential influence of organizational culture and climate on implementation strategy selection, implementation decision-making, and stakeholders’ perceptions of implementation strategies.

**Methods/design:**

This study is a mixed methods multiple case study of seven children’s social service organizations in one Midwestern city in the United States that compose the control group of a larger randomized controlled trial. Qualitative data will include semi-structured interviews with organizational leaders (*e.g*., CEOs/directors, clinical directors, program managers) and a review of documents (*e.g*., implementation and quality improvement plans, program manuals, etc.) that will shed light on implementation decision-making and specific implementation strategies that are used to implement new programs and practices. Additionally, focus groups with clinicians will explore their perceptions of a range of implementation strategies. This qualitative work will inform the development of a Web-based survey that will assess the perceived effectiveness, relative importance, acceptability, feasibility, and appropriateness of implementation strategies from the perspective of both clinicians and organizational leaders. Finally, the Organizational Social Context measure will be used to assess organizational culture and climate. Qualitative, quantitative, and mixed methods data will be analyzed and interpreted at the case level as well as across cases in order to highlight meaningful similarities, differences, and site-specific experiences.

**Discussion:**

This study is designed to inform efforts to develop more effective implementation strategies by fully describing the implementation experiences of a sample of community-based organizations that provide mental health services to youth in one Midwestern city.

## Background

Children in the U.S. continue to receive substandard mental health and child welfare services [1-4], partly because we do not understand how to effectively integrate evidence-based treatments (EBTs) into ‘real world’ service settings. Evidence-based treatments are seldom implemented, and when they are, problems with implementation can severely diminish their impact [5]. To improve the quality of care for children, EBTs will need to be complemented by evidence-based approaches to implementation [6]. Thus, the National Institutes of Health and the Institute of Medicine have prioritized efforts to identify, develop, refine, and test implementation strategies [7,8], which are defined as ‘systematic intervention processes to adopt and integrate evidence-based health innovations into usual care’ [9].

### State of the evidence for implementation strategies

While the health and mental health literatures describe many potentially promising implementation strategies [9], the evidence of their effectiveness remains imperfect [10-13]. Most strategies deliver only modest effect sizes [10], and are effective under some, but not all, conditions [14]. Passive strategies, such as disseminating educational materials and continuing education courses, may be useful in increasing knowledge, but are generally not sufficient to change provider behavior [15-18]. Training approaches that incorporate ongoing supervision and consultation can lead to therapist behavior change [15,18], but it is increasingly recognized that strategies need to move beyond focusing solely on provider level factors such as knowledge and expertise [19-21]. Indeed, implementing EBTs with fidelity does not always improve outcomes [22], suggesting that other barriers to quality service provision must also be addressed [23]. Implementation is a complex, multi-level process, and existing theoretical and empirical work suggests that ‘best practices’ in implementation would involve the planned use of multiple strategies to address barriers to change that can emerge at all levels of the implementation context [9,20,21,24-28]. There are a number of strategies that extend beyond the provider level [9]; however, in social services research, there are very few randomized studies that test the effectiveness of multi-level implementation strategies (for one exception, see [23]). More research is needed to develop effective ways of tailoring strategies to target implementation barriers [29] and to develop innovative strategies that are efficient, cost-effective, and robust or readily adaptable [30].

### The need for a better understanding of implementation as usual

Implementation scientists cannot develop these strategies ‘in a vacuum’ [31]; they must possess a thorough understanding of the service systems and organizational contexts in which these strategies will (hopefully) be adopted [32]. Hoagwood and Kolko warn that ‘it is difficult and perhaps foolhardy to try to improve what you don’t understand’ [31], and note that program implementers and services researchers are often unable to anticipate implementation challenges largely because the context of service delivery has not been adequately described. In other words, there is a need for a better understanding of usual care settings, and in particular, what constitutes ‘implementation as usual’.

Garland *et al.* acknowledge that ‘studies that “simply” characterize existing practice may not be perceived as innovative or exciting compared to studies that test new innovations’ [33]. However, these studies are ‘a necessary complement – if not precursor’ – to studies that will strengthen knowledge on the implementation of EBTs [31]. Indeed, an increased understanding of implementation as usual has the potential to identify leverage points for implementation, specify targets for improvement, and generate useful insights into the types of implementation processes that are likely to be successful in the real world.

At present, very little is known about the implementation processes that occur in usual care [31,33,34]. This highlights the need for descriptive studies that define the range and context of current implementation processes in relation to what is known about ‘best implementation practice’ [35], which (for the purpose of this study) is characterized as the planned use of multiple strategies to address barriers to change at various levels [20,26,28,36]. The current study addresses this need by leveraging a control group of a larger implementation trial that is not receiving an active implementation intervention. Using control groups to examine implementation as usual may yield critical information that can be used to improve the development of implementation strategies. This approach maximizes the use of research funding, illuminates implementation processes within control conditions that may be helpful in understanding the results of larger trials, and ultimately, avoids treating control conditions as ‘black boxes’ that are assumed to have no ‘action’ related to treatment and implementation decisions and processes. The last point constitutes a considerable advantage over studies that focus solely on outcomes obtained by control groups thought to represent ‘usual care’ without generating rich descriptions of what actually occurs in these settings. This study will describe four elements of these organizations that may play a role in determining implementation, service system, and clinical outcomes [37]: patterns of implementation strategy use, implementation decision-making, perceptions of implementation strategies, and organizational social context.

### Implementation strategy patterns

There is a paucity of descriptive data pertaining to basic contextual elements of implementation such as organizational operations, staffing patterns, and electronic technologies for tracking service visits in usual care settings [31]. Even less is known about implementation strategy patterns in children’s social service organizations. One exception is Schoenwald and colleagues’ examination of organizations’ use of training, supervision and evaluation [34]. Encouragingly, they found that training and supervisory practices were more or less ‘in line’ with the typical procedures in an effectiveness trial. However, there has yet to be a study that maps a fuller range of potential implementation strategies that extends beyond commonly used strategies such as training and supervision [9]. Thus, very little is known about the types of strategies employed, the frequency and intensity at which they are used, and the conceptual domains and levels of the implementation context that they target.

### Organizational decision-making related to implementation processes

Organizational leaders face tremendous challenges when it comes to determining which treatments will be implemented in their settings and how they will be implemented. As Ferlie notes, ‘implementation process is often emergent, uncertain, and affected by the local context and features of action’ [38]. It would be ideal if organizational leaders would base their decisions upon the latest theoretical and empirical findings; however, little is written about how organizational leaders approach implementation decision-making. In particular, we need to know more about whether and how organizational leaders use research related to management and implementation, and the conditions under which they may be more likely to use research [38]. Furthermore, there is a need for more insight into the types (*e.g*., summaries of implementation barriers and facilitators, reviews of implementation strategies), formats (*e.g*., statistical or narrative summaries), and sources (*e.g*., academics, peers from other organizations) of information that organizational leaders find most valuable when making decisions about how to implement EBTs. This will highlight the ways in which implementation research could be made more accessible to organizational leaders, and could inform the development of decision aids that could facilitate the identification, selection, and tailoring of implementation strategies.

### Stakeholders’ perceptions of the characteristics of implementation strategies

The characteristics of interventions may play a large role in determining whether or not they are adopted and sustained in the real world [26,39,40]. Rogers’ diffusion of innovations theory suggests that innovative treatment models will not likely be adopted unless they are: superior to treatment as usual; compatible with agency practices; no more complex than existing services; easy to try (and reject if it fails); and likely to produce tangible results recognizable by authorities [40,41]. Other potentially influential characteristics of interventions specified in theoretical models include the intervention source (*i.e*., the legitimacy of the source and whether it was internally or externally developed), evidence strength and quality, adaptability, design quality and packaging, and costs [26]. While these characteristics are often considered in relation to clinical interventions, they also readily apply to implementation strategies. In fact, a better understanding of stakeholders’ perceptions of implementation strategies may facilitate the process of identifying, developing, and selecting strategies that will be feasible and effective in the real world.

### Influence of organizational culture and climate on implementation processes

The conceptual and empirical literatures have underscored the importance of organizational factors such as culture and climate in facilitating or impeding the uptake of innovations [24,26,42-44]. ‘Organizational culture’ is what makes an organization unique from others, including its core values and its organizational history of adapting with successes and failures [42]. It involves not only values and patterns related to products and services, but also how individuals within an organization treat and interact with one another [42]. Glisson and colleagues write, ‘Culture describes how the work is done in the organization and is measured as the behavioral expectations reported by members of the organization. These expectations guide the way work is approached and socialize new employees in the priorities of the organization’ [43]. Thus, culture is passed on to new employees and is conceptualized as a rather stable construct that is difficult to change. ‘Organizational climate’ is formed when employees have shared perceptions of the psychological impact of their work environment on their own well-being and functioning in the organization [43].

More constructive or positive organizational cultures and climates are associated with more positive staff morale [45], reduced staff turnover [46], increased access to mental health care [47], improved service quality and outcomes [45,48,49], greater sustainability of new programs [46], and more positive attitudes toward EBTs [50]. Yet, it is less clear how culture and climate relate to implementation processes. Knowing more about this relationship would inform efforts to facilitate organizational change. For example, it may be that organizations with poor cultures and climates require more intensive implementation support in order to develop well-coordinated implementation plans that address relevant determinants of practice [51].

### Study aims

This mixed methods multiple case study addresses these gaps in knowledge related to implementation contexts and processes in children’s social service organizations through the following aims:

**Aim 1:** To identify and characterize the implementation strategies used in community-based children’s social service settings;

**Aim 2:** To explore how organizational leaders make decisions about which treatments and programs to implement and how to implement them;

**Aim 3:** To assess stakeholders’ (organizational leaders’ and clinicians’) perceptions of the effectiveness, relative importance, acceptability, feasibility and appropriateness of implementation strategies; and

**Aim 4:** To examine the relationship between organizational context (culture and climate) and implementation strategy selection, implementation decision-making, and perceptions of implementation strategies.

### Approach

Aim 1 will rely upon semi-structured interviews with organizational leaders (management and clinical directors) and document review to yield rich descriptions of the implementation strategies employed by seven agencies. This data will be compared to ‘best practices’ in implementation derived from existing theoretical and empirical work [11,13,15,18,36] to inform future work developing strategies in areas that are currently poorly addressed. It will also allow researchers and administrators to build upon ‘practice-based evidence’ and the strengths of ‘positive deviants’ (*i.e*., organizations that are consistently effective in implementing change despite a myriad of implementation barriers) [52,53].

Aim 2 will also use semi-structured interviews with organizational leaders and document review to generate new knowledge about how agency leaders use evidence and other sources of information to make decisions about implementation. Learning more about the type of information that organizational leaders seek, the sources they look to for that information, and the conditions under which they seek that information, may inform future work to make implementation science findings more accessible and ensure that implementation decision-making is based upon the best available theoretical and empirical knowledge in the field.

Aim 3 will utilize focus groups and an online survey to ensure that future work to develop and test implementation strategies will be informed by stakeholders’ (organizational leaders’ and clinicians’) perceptions about the types of strategies that are likely to be effective in the real world.

Aim 4 will examine how organizational social context (culture and climate) facilitates or hinders implementation by linking the data about strategy selection, implementation decision-making, and stakeholders’ perceptions of implementation strategies to organizations’ scores on a standardized measure of culture and climate [43].

### Guiding conceptual frameworks

The proposed study is informed by two conceptual frameworks: the consolidated framework for implementation research CFIR [26] and Grol and Wensing’s implementation of change model [36]. These models will be integrated in all stages of the research process, including conceptualization (*e.g*., selecting implementation processes on which to focus), data collection (*e.g*., using components of the conceptual models as interview questions and probes), analysis (*e.g*., determining how comprehensively organizations are addressing constructs essential to implementation success, comparing ‘implementation as usual’ to ‘best practices’), and dissemination (*e.g*., framing findings conceptually so that they will be comparable to other implementation studies).

The CFIR was developed for the purpose of serving as a common reference to the many constructs that have been identified as important to implementation success [26]. It identifies five major domains related to implementation, including: intervention characteristics, the outer setting, the inner setting, the characteristics of the individuals involved, and the process of implementation. Detailed definitions of the 39 constructs included in the CFIR can be found in the supplementary materials associated with that article [26]. It captures the complex, multi-level nature of implementation, and suggests that successful implementation may necessitate the use of an array of strategies that target multiple levels of the implementation context [9]. The CFIR has informed the semi-structured interview guide (see Additional file 1) by specifying specific probes for eliciting descriptions of implementation strategies across various ‘levels’. It will also be used to assess the comprehensiveness of organizations’ approaches to implementation. For example, an organization that focuses only on the ‘characteristics of individuals’ while neglecting other domains such as ‘intervention characteristics’ or the ‘inner setting’ would have a less comprehensive approach to implementation than an organization that addresses all three (or more) of those domains.

Grol and Wensing’s implementation of change model informs this research by specifying a process of implementation that begins with identifying problems or gaps in care, identifying ESTs or other best-practices, carefully planning the implementation effort, developing a proposal with targets for improvement or change, analyzing current performance, developing implementation strategies, executing the implementation plan, and continuously evaluating and (if necessary) adapting the plan [36]. The model provides a structure and a process to implementation that the CFIR lacks. It also emphasizes an important aspect of implementation ‘best practice, ’ namely, that while implementation processes may be complex, necessitating iterative and flexible approaches [54,55], they should be planned and deliberate rather than haphazard. The implementation of change model has also informed the development of the interview guide informing Aims 1 and 2.

## Methods/design

### Overview

This study employs a mixed methods multiple case study design, in which each participating organization (n = 7) is conceptualized as a ‘case’ [56,57]. Case studies are particularly helpful in understanding the internal dynamics of change processes, and including multiple cases capitalizes on organizational variation and permits an examination of how contextual factors influence implementation [58]. Leaders in the field have emphasized the importance of using case study and other mixed methods observational designs to develop a more nuanced, theoretically informed understanding of change processes [59-64]. The study relies upon the ‘sequential collection and analysis of qualitative and quantitative data, beginning with qualitative data, for the primary purpose of exploration and hypothesis generation, ’ or a QUAL → quan approach [64]. This serves the primary function of ‘development, ’ as collecting qualitative data in Aims 1 to 3 affords the opportunity to examine the impact of organizational context in Aim 4 [64]. It serves the secondary function of ‘convergence’ by using quantitative and qualitative data to answer the same questions in Aim 3 [64].

### Sample

The study will be conducted in the control arm of a U.S. National Institute of Mental Health funded randomized controlled trial (RCT) [65] of the Availability, Responsiveness, and Continuity (ARC) organizational implementation strategy [23,49,66], which affords a unique opportunity to study implementation as usual. The sample includes seven children’s social service organizations in a Midwestern city that reflect the characteristics of children’s mental health service providers nationwide [34] in that they are characterized by nonprofit organizational structures, they employ therapists that have master’s and bachelor’s degrees, and are comprised of a predominantly social work staff.

All participating organizations may not be currently implementing EBTs; however, they will likely be able to discuss strategies they have used to implement other clinical programs, services, or treatment models [46]. Thus, we will maintain an inclusive stance toward the types of programs and practices that organizations are implementing. This is warranted given that the primary scientific objective is to learn more about the processes and contexts of implementation rather than the particulars of implementing a specific EBT or class of EBTs.

While sampling logic should not be used in multiple case study research [57,67], seven cases are expected to be enough to ‘replicate’ findings across cases [57]. Yin writes that each ‘case’ (organization) is in essence treated as a separate study that either predicts similar results (literal replication) or predicts contrasting results but for anticipatable reasons (theoretical replication) [57]. In the present study, organizations with the worst cultures and climates may be expected to demonstrate similar implementation processes and perceptions of strategies (*i.e*., literal replication), whereas organizations with more positive cultures and climates may embrace a much different set of implementation processes and perceptions of strategies (*i.e*., theoretical replication).

### Data collection

The proposed study will rely upon qualitative data from semi-structured interviews (Aims 1, 2, and 4), document review (Aims 1, 2, and 4), and focus groups (Aim 3). Additionally, quantitative data from a project-specific survey being developed (described below) and the Organizational Social Context (OSC) measure [43] will be used to accomplish Aims 3 and 4 respectively (see Table 1).

**Table 1:** Data collection: measures and sources (QUAL → quan)

**Conceptual domains**	**Aims**	**Method**	**Measure source**	**Data source**	**Type of data**	**Sample size**
Implementation strategies	Aim 1	Semi-structured interview	Developed for Study	Key informants	QUAL	~21-35
		Document review	Agency	Agency documents	QUAL	NA
Implementation decision-making	Aim 2	Semi-structured interview	Developed for Study	Key informants	QUAL	~21-35
		Document review	Agency	Agency documents	QUAL	NA
Stakeholder perceptions regarding strategy characteristics	Aim 3	Focus Groups	Developed for Study	Clinical staff	QUAL	7 groups w/ ~4-8 participants each
		Survey	Developed for Study	Managerial & Clinical staff	quan	~100 participants
Organizational context	Aim 4	Survey (Glisson *et al*., 2008)	Glisson & colleagues	Clinical staff	quan	7 organizational profiles; 10 programs; ~100 participants

### Qualitative data collection

#### ***Semi-structured interviews***

Semi-structured interviews will be conducted with organizational leaders (*e.g*., management and clinical supervisors) from each participating organization. The interviews will explore the implementation strategies their agencies have employed within the past year (Aim 1) and their approach to implementation decision-making (Aim 2). Interviews will be conducted by the lead author and will be structured by an interview guide (Additional file 1) informed by a review of implementation strategies [9] and the guiding conceptual models [26,36]. Specifically, the interview guide contains questions and prompts that will encourage participants to consider the implementation strategies that their organization has employed at multiple levels of the implementation context as specified by the CFIR [26] and the Powell *et al.* taxonomy [9] (*e.g*., asking if their organization used strategies related to the intervention, the policy or inter-organizational level, and the organization’s structure and functioning in addition to more commonly considered individual-level and process-level strategies). Through the process of snowball sampling [68], each participant will be asked to identify other employees who possess the requisite knowledge and experience to inform the study’s objectives. It is estimated that each organization will identify between three and five key informants, resulting in approximately 21 to 35 total interviews. Many agencies may not have more than this number of individuals who have direct knowledge of the use of implementation strategies [69], and more importantly, the decision-making processes surrounding implementation.

Guest and colleagues emphasize that very small samples can yield complete and accurate information as long as the respondents have the appropriate amount of expertise in the domain of inquiry [70]. Further, a main benefit of the multiple case study design is obtaining different sources of information that will be used to triangulate the interview data [57,64]. Interviews will last 60 to 90 minutes and will be digitally recorded. Immediately following each interview, the interviewer will complete field notes that will capture the main themes of the interview and any information that is pertinent to the study aims [71,72]. Interviews and field notes will be transcribed, and entered into NVivo, version 10, for data analysis.

#### ***Document review***

The study will also involve a review of publically available and organization-provided documents. Organizational leaders will be asked to provide access to any documents that describe and formalize implementation processes. For example, these processes may be captured in notes from a board meeting in which the implementation of a new program or practice was discussed, or in an organization’s response to a request for proposals that seeks funding for a particular training or implementation related resource. Other documents may include (but are certainly not limited to) formal implementation or quality improvement plans, annual reports, and program manuals. These sources will serve to augment or triangulate interview respondents’ descriptions of implementation strategies and decision-making processes. With permission from the organizations, potentially useful documents will be obtained and entered into NVivo, version 10, for analysis.

#### ***Focus groups interviews***

Focus groups involving approximately four to eight clinicians (or direct care staff members) will be conducted in each participating organization to capture the depth and nuances of their perceptions of strategies. The number of participants per focus group is consistent with Barbour’s recommendation of a minimum of three or four participants and a maximum of eight [73]. The number of focus groups (one per agency) is appropriate because the relatively homogenous population (*e.g*., clinicians at a given agency) and the structured and somewhat narrow scope of inquiry reduces the number of individuals needed to reach saturation [70]. Further, the quantitative data will serve to triangulate the focus group data [57,64], reducing the need for a larger sample size. The focus groups will be conducted by the first author and a research assistant. The interview will be guided by a structured interview guide (Additional file 2) informed by a conceptual taxonomy of implementation outcomes [74]. Participants will be asked to discuss the implementation strategies that they have used at their organization, and the facilitator(s) will record each strategy mentioned on a whiteboard so that all participants can see the running list. Additional strategies drawn from the literature may be listed if the participants focus on a relatively narrow range of strategies. Participants will then be asked to reflect upon the effectiveness, acceptability, feasibility, and appropriateness of the listed strategies. Although the primary purpose of the focus group interviews is to assess participants’ perceptions of various implementation strategies, it is also possible that these individuals will provide information about implementation strategies used at their organization that were not captured in the semi-structured interviews with organizational leaders.

Each focus group will last approximately 60 to 90 minutes and will be digitally recorded. As with the individual interviews, the interviewer will complete field notes following the focus groups that will document the main themes of the session and any observations pertinent to the study aims. The interviews and the field notes will be transcribed and entered into NVivo, version 10, for analysis.

### Quantitative survey data

#### ***Survey of stakeholders’ perceptions of implementation strategies***

A project-specific self-administered web-based survey will be developed to assess stakeholders’ perceptions and experiences with specific implementation strategies. The implementation strategies included in the survey will be generated from the qualitative work in Aims 1, 2, and 3 and a published ‘menu’ that describes 68 distinct implementation strategies [9]. In order to ensure a relatively low burden to respondents, it is unlikely that more than 40 strategies will be included. Decisions about the inclusion of strategies will be driven by the qualitative analysis (*i.e*., using the strategies mentioned by organizational leaders and clinicians), while attempts will be made to include strategies that address a number of different targets as specified in the CFIR [26].

It should also be noted that the Powell and colleagues’ compilation includes a number of strategies that could not be reasonably adopted by the participants of this study (*e.g*., ‘centralize technical assistance’) [9], and those strategies will be eliminated. The survey will also be informed by a conceptual taxonomy of implementation outcomes [74] and other existing surveys drawn from implementation science measures collections [75,76]. In addition to basic demographic questions, stakeholders will be asked whether or not they have experienced each included implementation strategy (yes or no) and will then rate each strategy (using a Likert-style scale) on the following dimensions: ‘effectiveness’ and ‘relative importance’ (*i.e*., How well did it work and how important was it relative to other strategies?), ‘acceptability’ (*i.e*., How agreeable, palatable, or satisfactory is the strategy?), ‘feasibility’ (*i.e.*, the perception that the strategy has been or could be successfully used within a given setting), and ‘appropriateness’ (*i.e*., the perceived fit, relevance, or compatibility of the strategy with the setting). This survey will be administered via an email with a link to the online survey, and will be pilot tested to ensure face-validity and ease of use.

#### ***Organizational social context (OSC) survey***

The OSC is a standardized measure that assesses organizational culture, climate, and work attitudes (the latter of which is not being used for the current study) using 105 Likert-style items [43]. Culture is assessed in terms of an organization’s level of ‘rigidity’ (centralization, formalization), ‘proficiency’ (responsiveness, competence), and ‘resistance’ (apathy, suppression). The ‘best’ organizational cultures are highly proficient and not very rigid or resistant, while the ‘worst’ cultures are not very proficient and are highly rigid and resistant to change or new ideas. Climate is assessed with three second-order factors: ‘engagement’ (personalization, personal accomplishment), ‘functionality’ (growth and achievement, role clarity, cooperation), and ‘stress’ [43]. The ‘best’ organizational climates are described as being highly engaged, highly functional, and low in stress [43]. Cronbach’s alphas for the OSC subscales (rigidity, proficiency, resistance, stress, engagement, functionality) range from 0.78 to 0.94. The OSC will be administered on site, and a research assistant will assure respondents that their responses will remain confidential.

### Data analysis

#### ***Qualitative data analysis***

Qualitative data from semi-structured interviews, document review, and focus groups will be imported and analyzed (separately) in NVivo using qualitative content analysis [77-80], which has been used successfully in similar studies [81-83]. Content analysis enables a theory driven approach, and an examination of both manifest (*i.e*., the actual words used) and latent (*i.e*., the underlying meaning of the words) content [72]. Accordingly, analysis will be informed by the guiding conceptual models, with additional patterns, themes, and categories being allowed to emerge from the data [72,84]. The first author and a doctoral student research assistant will independently co-code a sample of the transcripts to increase reliability and reduce bias [72,85]. Both coders will participate in a frame-of-reference training to ensure a common understanding of the core concepts related to the research aims [82]. Disagreements will be discussed and resolved through consensus. Initially, the coders will review the transcripts to develop a general understanding of the content. ‘Memos’ will be generated to document initial impressions and define the parameters of specific codes. Next, the data will be condensed into analyzable units (text segments), which will be labelled with codes based on *a priori* (*i.e*., derived from the interview guide or guiding theories) or emergent themes that will be continually refined and compared to each other. For instance, the implementation of change model [36] will be used to develop *a priori* codes such as ‘identifying programs and practices’ or ‘planning’ related to implementation decision-making. The CFIR [26] will be used in a similar fashion by contributing *a priori* codes that will serve to distinguish different types of implementation strategies, such as strategies that focus on the ‘inner setting’ or the ‘outer setting’. Finally, the categories will be aggregated into broader themes related to implementation strategy patterns, implementation decision-making, and stakeholders’ perceptions of strategies.

The use of multiple respondents is intentional, as some individuals may be more or less knowledgeable about their organization’s approach to implementation; however, it is possible that participants from a given agency may not endorse the use of the same strategies [86]. The approach to handling such ‘discrepancies’ will be one of inclusion, in that each unique strategy endorsed will be recorded as ‘in use’ at that agency (for an example of this approach, see Hysong *et al.*[82]). If participants’ responses regarding strategies vary widely within a given organization, it may be indicative of a lack of a coherent or consistent strategy [86]. The use of mixed methods and multiple sources of data will allow us to make sense of reported variation in strategy use by affording the opportunity to determine the extent to which these sources of data converge [57,64,86,87]. The use of multiple respondents and different sources of data also reduces the threat of bias that is sometimes associated with the collection of retrospective accounts of phenomena such as business strategy [69].

#### ***Quantitative data analysis***

The developed survey capturing stakeholders’ perceptions of implementation strategies will yield descriptive data that will augment the qualitative data from semi-structured interviews, document review, and focus groups. In the cross-case analysis, this data will be compared to determine differences and similarities between cases. Data will also be pooled across all seven cases to reveal an overall picture of strategy use, as well as perceived effectiveness, relative importance, acceptability, feasibility, and appropriateness of implementation strategies.

Results from the OSC measure will be analyzed and interpreted in consultation with its developer according to procedures described by Glisson *et al*. [43]. Scoring will be completed at the University of Tennessee’s Children’s Mental Health Services Research Center, including the generation of internal reliability estimates (alpha), agreement indices for organizational unit profiles, and t-scores for culture and climate. The resulting organizational profiles can be compared to norms from a nationwide sample of 1,154 clinicians in 100 mental health clinics, which affords the opportunity to determine the generalizability of study findings beyond the selected sites. The OSC data will serve to characterize the organizations’ culture and climate in individual case descriptions. Additionally, organizations will be stratified by their OSC profiles in order to differentiate more positive cultures (highly proficient and not very rigid or resistant) and climates (highly engaged, highly functional, low stress) from less positive cultures (low proficiency, highly rigid and resistant) and climates (low engagement and functionality, high stress) [43]. Qualitative results will then be categorized according to those OSC profiles to determine whether strategy patterns, approaches to decision-making, and perceptions of strategies vary by organizational culture and climate.

#### ***Mixed methods analysis***

As previously mentioned, the structure of this study is QUAL → quan, meaning that qualitative methods precede quantitative and that they are predominant [64,88]. This serves the primary function of ‘development,’ as collecting qualitative data in Aims 1 to 3 affords the opportunity to examine the impact of organizational context in Aim 4. It also serves the function of ‘convergence’ by using quantitative and qualitative data to answer the same question in Aim 3 [64].

The processes of ‘mixing’ the qualitative and quantitative data flow directly from these functions. To serve the function of ‘development, ’ the quantitative data on organizational social context [43] is connected with the qualitative and quantitative results from Aims 1 to 3 regarding implementation strategy use, implementation decision-making, and stakeholder perceptions of implementation strategies [64]. Assuming there is a meaningful relationship between organizational social context and the data from Aims 1 to 3, this can be shown in a joint display [88] that categorizes the themes emerging from the qualitative and quantitative data based upon the OSC profiles [43] as described above. For example, a separate table may be used to show how implementation strategy patterns differ based upon organizational social context. Examples of this approach can be found in Killaspy *et al*. [89] and Hysong *et al*. [82], and are also detailed in Creswell and Plano-Clark’s methods book [88].

To serve the function of ‘convergence, ’ the qualitative data and quantitative data will be merged in order to answer the same question, which for Aim 3 is, ‘What are implementation stakeholders’ perceptions of implementation strategies’? These data are merged for the purpose of triangulation; in this case, to use the quantitative data from the stakeholder perceptions survey to validate and confirm the qualitative findings from the focus-group interviews. Once again, this process can be depicted through a table placing qualitative themes side by side with the quantitative findings to show the extent to which the data converges [88].

It is worth noting that the approaches to ‘mixing’ qualitative and quantitative data will be used at both the case-level and the cross-case level (as described below).

#### ***Cross-case analysis***

A primary benefit of a multiple case study is the ability to make comparisons across cases. The proposed study will utilize cross-case synthesis [57], which treats individual cases as separate studies that are then compared to identify similarities and differences between the cases. This will involve creating word tables or matrices that will display the data according to a uniform framework [57,84]. For example, data from the first three aims (strategy patterns, implementation decision-making, and stakeholder perceptions) will be categorized based upon their OSC profiles [43] in Aim 4. This approach will be used to compare across cases for each of the proposed aims, allowing for meaningful similarities, differences, and site-specific experiences to emerge from the data [56,57].

### Limitations

A number of limitations should be considered. There is some concern that the organizations in the sample will not be comparable since they will not all be implementing the same programs and practices. There are several protections against this danger. First, while there is evidence to suggest that specific programs and practices will require unique implementation strategies *e.g*., [90], implementation strategies can also be viewed as more general components of an organization’s infrastructure [34]. In fact, this view of implementation strategies may become more salient as we begin to shift the focus away from implementing solitary practices and toward fostering evidence-based systems and “learning organizations” capable of implementing a number of EBTs well [91]. Obtaining descriptive data about the types of implementation strategies that organizations are currently using is a first step toward determining which strategies may need to be routinized in organizations and systems of care. Second, while they may not all be implementing the same interventions, the organizations in this sample are comparable in terms of client need, service provision, funding requirements, and other external or ‘outer setting’ factors [26]. Third, programs and practices can be compared in meaningful ways based upon their characteristics [39,40,92].

The cross-sectional nature of the data will not reveal how implementation processes change over time. Additionally, recall bias may limit the accuracy of participants’ memories of implementation processes. The use of multiple informants and data sources (*i.e*., triangulation) will increase the validity of findings and minimize the threat of this bias [57,58].

A final challenge is the lack of existing surveys that can assess stakeholder perceptions of strategies; however, the web-based survey will be informed by theories related to the intervention characteristics associated with increased adoption [26,39,40], related surveys [75], a taxonomy of implementation outcomes [74], and other emerging measurement models *e.g*., [93].

### Trial status

The Institutional Review Board at Washington University in St. Louis has approved all study procedures. Recruitment and data collection for this study began in March of 2013.

## Discussion

Improving the quality of children’s social services will require ‘making the right thing to do, the easy thing to do’ [94] by providing organizational leaders and clinicians with the tools they need to provide evidence-based care. In order for this to be accomplished, there is much we need to know about the approaches to implementation that routinely occur, the ‘on the ground’ perspectives of organizational stakeholders regarding the types of implementation strategies that are likely to work, and the ways in which organizational context impacts implementation processes. This study represents a novel approach to studying implementation as usual in the control group of an implementation RCT. By shedding light on ‘implementation as usual’ in children’s social service settings, this study will inform efforts to develop and tailor strategies, propelling the field toward the ideal of evidence-based implementation.

## Abbreviations

ARC: Availability Responsiveness and Continuity; CFIR: Consolidated Framework for Implementation Research; EBTs: Evidence-Based Treatments; NRSA: National Research Service Award; NIMH: National Institute of Mental Health; OSC: Organizational Social Context; RCT: randomized controlled trial; QUAL: qualitative (dominate method); quan: quantitative (subordinate method).

## Competing interests

The authors declare that they have no competing interests.

## Authors’ contributions

BJP is the principal investigator of the study. BJP generated the idea and designed the study, drafted the manuscript, and approved all changes. EKP is the primary mentor on BJP’s F31 award from the National Institute of Mental Health and the award from the Doris Duke Charitable Foundation. CAG is the principal investigator and EKP is the co-principal investigator of the ARC RCT that provides the context for the current study. CAG is the developer of the OSC survey, and he and his colleagues from the Children’s Mental Health Services Research Center at the University of Tennessee, Knoxville will assist with the analysis and interpretation of that data. EKP, CAG, PLK, RR, RCB, BPS, CRC, and LAP provided input into the design of the study. All authors reviewed and provided feedback for this manuscript. The final version of this manuscript was vetted and approved by all authors.

## Supplementary Material

Additional file 1 Semi-Structured Interview Guide. Click here for file

Additional file 2 Focus Group Interview Guide. Click here for file
