# Supplementary material for: A mixed methods multiple case study of implementation as usual in children’s social service organizations: study protocol
Source: Implement Sci. 2013 Aug 20;8:92. doi: 10.1186/1748-5908-8-92 (PMC3751866; doi:10.1186/1748-5908-8-92)
Supplement: Additional file 1 — Semi-Structured Interview Guide. [file 1748-5908-8-92-S1.pdf]

Semi-Structured Interview Guide

Thank you for taking the time to meet with me. As you are aware, this study focuses on implementation and quality improvement in mental health. I am going to ask you a series of questions regarding your organization's efforts to improve care by implementing new programs. I'm interested in how your organization makes decisions about *what* programs and practices to implement and *how* they should be implemented (i.e., the deliberate processes that should be used to integrate a particular program or practice). Thus, I will ask you a series of questions about your decision making processes as well as the specific implementation strategies that your organization has used over the course of the last year or so. I will ask you to be as specific as possible regarding the content of the implementation strategies that you used. For instance, though it would be helpful to know that your organization trains clinicians in a new program, it would be more helpful to know the basic components of that training and details about its frequency, duration, and intensity. I will also remind you throughout the interview to be explicit about the programs and practices you are referring to (if there are more than one), as well as the different stages or phases of implementation that you are referring to (e.g., pre-implementation vs. sustainability monitoring). It is expected that there will be details that you are not aware of or can't recall, and it also may be the case that another organizational leader or employee is better equipped to provide this information. Thus, after the interview, I will ask you if there are other people within your organization that I should also interview if given the opportunity. We are thrilled to be able to learn from your experiences, and we hope that this study contributes to future research developing implementation strategies that will be more effective and helpful to organizational leaders like yourself. Thus, as you reflect upon your experiences implementing new programs and practices, please be as frank as possible about what works, what doesn't, and the pragmatic constraints that you face as a leader in children's mental health.

**Question**	**Possible Prompts**
Program(s) or Practice(s) Implemented	
-Can you tell me about a new practice or set of practices that your organization has implemented in the past year or so? If your organization has implemented a wide range of practices, I will ask you to focus on the implementation of a single practice or 2-3 practices with similar characteristics.	

Implementation Decision Making (EBT or Practice Related)	
-How did your organization decide to implement the practice(s) or program(s)?	
-What types of information or "evidence" did you seek to inform your decisions?	
-Which types of information or "evidence" were most important to your decision making process?	
Implementation Decision Making (Processes and Strategies)	
-After you decided to implement the practice or program, what factors were considered when you thought about how to implement it within your organization?	-How did you plan for implementation?<br>-Who was involved in this process?<br>-Was there a formal evaluation of organizational performance prior to and after implementation?<br>-Did your organization rely upon any formal models of implementation or quality improvement?<br>-Was there a formal plan developed and documented?<br>-If not, what were the guiding principles that informed your implementation processes?<br>-Were there any plans for assessing or reevaluating the implementation process?
-What types of information or "evidence" did you seek to inform your decision about how to approach implementation?<br>-Where did you seek this information?	
-Which types of information were most important in guiding your decisions about how to approach implementation?	
Implementation Strategies	
-How did the organization implement the new program(s) (i.e., what implementation strategies did you use)?<br>-Has your organization used any additional strategies to implement programs and practices that we have not discussed today?	*Probe for specifics of implementation strategies/processes.* Could provide specific examples from Powell and colleagues' compilation of implementation strategies to sensitize participants to the notion of implementation strategies.[15]<br><br>Did you adopt strategies that addressed

-Did you find that different practices required the use of unique implementation strategies?	and of the following based upon Damschroder and colleagues conceptual framework.[22]<br><br>-Did your organization use any implementation strategies that were directed at the intervention itself? For example, was the program adapted in any way to fit your client base?<br><br>-Did your organization use any strategies to address external policies or incentives that might be relevant to implementing the intervention?<br><br>-Did your organization collaborate with other organizations in any way to implement the intervention?<br><br>-Did your organization use any implementation strategies at the funding level? For instance, strategies to access new funding for the implementation effort?<br><br>-Did your organization use any strategies that were directed at changing its structure or functioning in ways that would facilitate the implementation of the intervention?<br><br>-Did your organization use any strategies that were directed at providers? For example, training and supervision are provider-focused implementation strategies.<br><br>-Did your organization use any implementation strategies that were directed at your clients?<br><br>-Did your organization use any strategies that were more process-oriented, such as planning, reflecting, or evaluating the implementation effort?

The Perceived Effectiveness of Implementation Strategies (i.e., "Practice-Based Evidence")	
-In your experience, have some implementation strategies been more or less effective than others? If so, which strategies have been particularly effective? Ineffective?	
-Have you found certain implementation strategies to be more acceptable, feasible, and sustainable than others?	
Wrap-Up (Other)	
-What advice would you give to others who might lead their organizations in the implementation process?	
-Is there anything else that you would like to share related to your experiences with implementation or anything else that we have discussed today?	
