# Supplementary material for: A mixed methods multiple case study of implementation as usual in children’s social service organizations: study protocol
Source: Implement Sci. 2013 Aug 20;8:92. doi: 10.1186/1748-5908-8-92 (PMC3751866; doi:10.1186/1748-5908-8-92)
Supplement: Additional file 2 — Focus Group Interview Guide. [file 1748-5908-8-92-S2.pdf]

Focus Group Interview Guide

Thank you so much for taking the time to participate today. As you are aware, this research project focuses on efforts to improve the quality of children's mental health care by implementing evidence-based programs and practices. We are interested in your experiences with implementation process. The majority of this focus group will focus on implementation strategies, or the deliberate processes by which your organization has attempted to integrate a particular program or practice. Thus, a series of questions will be posed to give us a common reference by which to discuss your experiences and perceptions relative to specific implementation strategies. Your "practice-based evidence" will inform future research focused on the development of implementation strategies that will hopefully make implementation and service delivery more effective. This will be a free-flowing discussion, so please feel free to share your thoughts, questions, and concerns throughout the process.

**Question**	**Possible Prompts and Other Instructions**
Program(s) or Practice(s) Implemented	
-Could you please talk about something that [insert organization] has tried to implement in the past year or so?	Facilitator could mention specific programs or practices that were discussed as being implemented in earlier phases of this research (i.e. in semi-structured interviews).
Implementation Strategies	
-How did your organization attempt to implement this program or practice (i.e., what specific strategies were used)?	*Probe for specifics of implementation strategies/processes.*<br><br>Have group discuss a range of implementation strategies, writing them down so that they are visible to the entire group.<br><br>Could provide specific examples from Powell and colleagues' compilation of implementation strategies and/or a list of strategies that were identified by organizational leaders in that agency in order to sensitize participants to the notion of implementation strategies.
Relative Importance (Effectiveness) of Implementation Specific Implementation	

Strategies	
-Of the strategies you listed, could you talk about those that have been most critical to the successful implementation of a program or practice?<br>-Were some strategies more or less useful depending upon the stage of implementation (e.g., early vs. late, planning vs. sustaining)?<br>-Why were they so important?	
-Conversely, have some of the strategies listed been ineffectual or simply less helpful to you?<br>-Why was this the case?	
Acceptability	
-Are any of the strategies listed simply more agreeable, palatable, or satisfactory to you?	
Feasibility	
-Did any of the strategies listed sound good *in theory*, but prove to be not as helpful *in practice*?	
Appropriateness	
-Do some of the strategies listed simply fit your organization better than others?	
Wrap-Up	
-Are there other things that you would like to share with me related to your experience of implementation and implementation strategies?	
